# Ancient Myths and Avian Pestilence

**DOI:** 10.3201/eid1108.AC1108

**Published:** 2005-08

**Authors:** Polyxeni Potter

**Affiliations:** * Centers for Disease Control and Prevention, Atlanta, Georgia, USA

**Keywords:** Art and science, emerging infectious diseases, migratory birds, waterfowl, ornithes, avian influenza, west nile virus, stymphalian birds, labors of herakles

**Figure:**
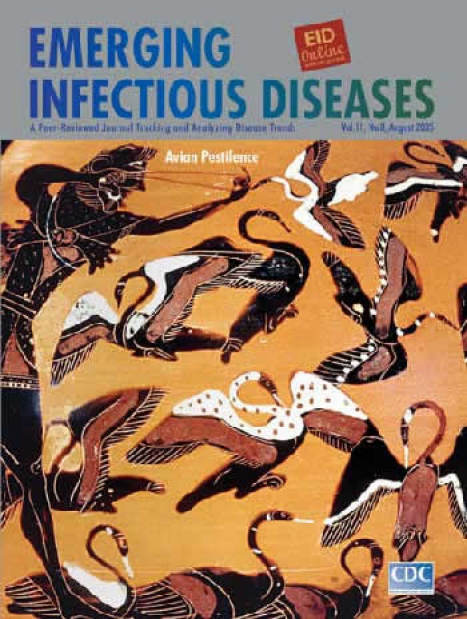
**Herakles and the Stymphalian Birds (detail) (circa 6th century BC).** Athenian black-figured amphora (40.6 cm). The British Museum, London, UK

"These birds are the size of a crane and are like the ibis, but their beaks are more powerful, and not crooked like the ibis," wrote ancient Hellene traveler and writer, Pausanias (*1*). He was referring to large flocks of metal-clawed ornithes, which according to legend, roosted in the dense marshes around Lake Stymphalis in Arcadia, ravaging crops and the livelihood of neighboring villages (*2*). This scourge, he speculated, was not local. "The Arabian Desert breeds, among other wild creatures, birds…which are quite as savage against men as lions or leopards…. These fly against those who come to hunt them, wounding and killing them with their beaks" (*1*).

The flesh-eating predators further terrorized local inhabitants by dispatching against them razor-edged feathers like arrows. "All armor of bronze or iron that men wear is pierced by the birds," elaborated Pausanias (*1*). Pets of Ares, god of war, these birds were a public menace too great for the community to control, a challenge finally assigned, along with other "labors," to strongman of all time, Herakles.

Son of Zeus and mortal Alcmene, Herakles might have enjoyed the privileged life of a demigod. But, victimized by Zeus' jealous wife, Hera, he endured a mortal lot of labor and hardship, punctuated by periods of madness and aberrant behavior. Strong, resourceful, and gifted with magical defenses, he had to struggle, nonetheless, against nature that was deadly, unpredictable, and arbitrary. During his celebrated labors, he battled vicious beasts (among them Kerberos, the guard of Hades) and cleaned out the infamous Augean Stables, which housed the filthiest herd of cattle in Hellas. To attain immortality, he performed, as penance for his misdeeds, arduous service to the community, using his unparalleled strength to support his fellow humans.

The thick marsh habitat of the Stymphalian birds worked against Herakles. His bow and arrows failed, for he could neither see nor reach the birds through the dense vegetation. Only asked to drive them away, he abandoned efforts to eliminate the birds; instead, he conned them into leaving the area on their own. With a pair of krotala (metal rattles) made by Hephaestus, god of the forge, Herakles frightened the birds out of their refuge and chased them as they flew east to the Isle of Ares in the Black Sea.

The detail on this month's cover comes from a black-figured amphora, a ceramic vase popular in ancient Athens in the 6th century BC. Such vases were made of iron-rich clay and decorated with black silhouettes in mythical heroic scenes. Illustrations were incised and painted with a slip (liquid clay), which turned black during firing without oxygen (*3*).

The scene is full of action but contains no background clues. Hellenic myths focus on the here and now and its terrifying uncertainties and dilemmas. They address human concerns, not philosophical conceit. Their narrative blurs the boundaries of history and legend as heroes cross back and forth from fantasy to reality, often operating in geographic locations that can never quite be verified on a map.

Herakles cuts a powerful figure as he leans forward, aiming a sling at the birds. His body, draped with the impenetrable hide of the Nemean Lion, a trophy from his first labor, forms a barrier against the flock. The birds scatter in disarray, not laden with metal as the myth prescribes, but confused, half resting at the foot of the hero, half flapping their wings against each other, compromised by the lack of cover. These are beautiful birds, dotted and striped, with elegant long necks turned defensively inward. Yet, in some versions of the Stymphalian labor, the birds are harpies—half metal-feathered ornithes, half human heads with bronze beaks.

Herakles probably wished he had not stopped at chasing these Arabian birds away from Arcadia for, even in the small world of antiquity, geographic migration ruled. The birds surfaced again, during his sail with Jason and the Argonauts in search of the Golden Fleece, to be chased away again, this time by the sons of the North Wind.

Flawed humanity tested by overwhelming challenges rings true today. Heroic figures battling great odds excite our collective imagination. And public challenges (waste pollution out of control, avian pestilence) have changed little. Waterfowl, a benign species, were demonized in the Stymphalian myth, their hideous mien likely borne of human fear and helplessness, for who knows what pestilence they had inflicted on the community around the lake. And each time those birds flew to a new place, they had contact with other birds and opportunities for genetic reassortment, redistribution, and modification of pathogens throughout the migratory route.

Resistant to slings and arrows and prone to long-distance migrations, birds such as the ones on this cover's amphora persist beyond our ancestors' morbid imaginations. Not because of mythical metal paraphernalia but for their explosive potential as natural reservoirs and amplifying hosts of pathogens. Viremic migratory birds acting as introductory hosts may have brought West Nile virus to the Western Hemisphere, perhaps by infecting ornithophilic mosquitoes, which may have infected amplifying hosts and eventually humans (*4**,**5*).

Migratory waterfowl (ducks, geese) also carry flu viruses in their intestines and shed them in their secretions and excretions. As these waterfowl migrate around the globe, they introduce new flu strains into domestic poultry and swine. These strains can then amplify and mutate close to human populations, increasing the risk that the virus will recombine with local human strains to form a new virus with pandemic potential. Like the legendary harpies, these new strains, half human half avian, pose an immense public health challenge. We now know more about bird pestilence. West Nile virus infection and avian flu are just as ominous as razor-edged feathers. And while Herakles had krotala from the gods, we must work with human tools: repellants and pesticides, vaccines (*6*), antiviral drugs, or medical isolation and quarantine.
